# Long-term pH Alterations in the Periradicular Area Following the Application of Calcium Hydroxide and MTA

**DOI:** 10.30476/DENTJODS.2020.86534.1195

**Published:** 2021-06

**Authors:** Noushin Yazdanpanahi, Ali Behzadi, Maryam Zare Jahromi

**Affiliations:** 1 Dental Graduate Student, School of Dentistry, Isfahan (Khorasgan) Branch, Islamic Azad University, Isfahan, Iran; 2 Postgraduate Student, Dept. of Pediatric Dentistry, School of Dentistry, Isfahan (Khorasgan) Branch, Islamic Azad University, Isfahan, Iran; 3 Dept. of Endodontics, School of Dentistry, Isfahan (Khorasgan) Branch, Islamic Azad University, Isfahan, Iran

**Keywords:** pH, Calcium Hydroxide, Mineral Trioxide Aggregate, Intracanal Medicament

## Abstract

**Statement of the Problem:**

A rise in pH and the presence of calcium ions play an important role in prevention or management of external root resorption.

**Purpose:**

This study assessed the long-term pH alterations in the periradicular area following the application of calcium hydroxide (CH) and mineral trioxide aggregate (MTA) intracanal medicaments.

**Materials and Method:**

This *in vitro*, experimental study evaluated 45 single-canal extracted human teeth. After decoronation and root canal instrumentation, defects (3×3×1mm) were created in the middle third of the roots. Following smear layer removal, the root surface (except for the defect) was sealed with nail varnish. Five teeth served as negative controls and were filled with distilled water. The remaining 40 teeth were randomly divided into two groups (n=20) for application of MTA and CH as intr-acanal medicaments. Periapical radiographs were obtained to ensure optimal quality of obturation. After coronal sealing with glass ionomer, the teeth were incubated at 37°C, and their pH was measured at 1 and 2 weeks, and 1 and 3 months, using a pH-meter. Data were analyzed using one-way ANOVA, Tukey’s test and Bonferroni adjustment.

**Results:**

The mean pH was significantly higher in CH group at 1 and 2 weeks (*p*< 0.01) but no difference was noted at 1 and 3 months (*p*= 0.52). The mean pH in both groups was significantly higher at 2 weeks compared with other time points (*p*< 0.05).

**Conclusion:**

CH may be preferred for use in the first weeks following the initiation of root resorption to provide a high pH. MTA can be later applied to maintain the high pH for a longer period of time without the need for medicament exchange.

## Introduction

The ultimate goal of endodontic treatment is complete elimination of bacteria and their byproducts as well as the pulpal residues from the infected canals, complete disinfection, and subsequent root canal filling [ 1 ]. Efficient chemomechanical preparation is an imperative step for elimination or reduction of bacteria in the root canal system. However, due to the complexity of the root canal system, over 50% of the root canal walls remain uninstrumented after root canal treatment. Thus, intracanal medicaments are commonly applied in combination with chemomechanical root canal preparation for disinfection of necrotic root canals [ 2 - 3 ]. 

Calcium hydroxide (CH) and mineral trioxide aggregate (MTA) favorably increase the root surface pH, maintain a high pH, and release calcium ions to exert antimicrobial activity and induce dentinogenesis by induction of growth factor release [ 2 ]. Different types of intracanal medicaments including phenols, aldehydes, halides, steroids, CH, antibiotics, and a mixture of medications are used for root canal disinfection. Hydrated CH with a molecular weight of 74.08 is commonly used in dentistry. In presence of water, it breaks down into Ca^2+^ and OH^-^ ions. Thus, when used as intracanal medicament, the calcium and hydroxyl ions penetrate into dentinal tubules [ 4]. It has low water-solubility and its solubility further decreases by temperature rise [ 5 - 7]. In endodontics, CH is used for vital pulp therapy, management of internal root resorption or traumatic root perforation, and root filling in primary teeth [ 8- 13]. 

The mechanism of action of CH directly depends on the dissolution of calcium and hydroxyl ions. CH can increase the local pH to 12.5. In addition to favorable intracanal effects, CH releases calcium and hydroxyl ions and accelerates periapical healing as such [ 14]. The alkaline pH of CH neutralizes the lactic acid produced by osteoclasts in the periapical region and prevents demineralization as such [ 15]. CH also activates the alkaline phosphatase and enhances hard tissue formation. Moreover, the gradual release of calcium from CH activates the growth factors required for hard tissue formation [ 16]. Thus, CH is the medication of choice for prevention and treatment of inflammatory root resorption. 

MTA was first used as a root-end filling material composed of Portland cement [ 17]. MTA is hydrophilic cement, which transforms into a colloidal gel after being mixed with water [ 18]. It has a pH of 10.2 at the time of mixing, which increases to 12.5 in the process of setting, and remains constant for 3 h [ 19 - 20 ]. Its final setting time is about 4 h [ 19 ]. MTA is commonly used as a root-end filling material and for perforation repair, vital pulp therapy, and formation of apical plug in necrotic and open-apex teeth [ 21 ]. Optimal sealability, favorable biocompatibility, optimal adaptation due to slight setting expansion, osteoconductivity and radiopacity are among the favorable properties of MTA [ 22 ]. Unlike CH, MTA does not undergo wear or resorption following exposure to periradicular tissue. Continuous exposure to water and heat in the oral cavity further contributes to its final setting [ 23 ]. MTA contains calcium oxide. When mixed with water, it forms CH and releases hydroxyl ions, which increase the pH and exert antimicrobial and antifungal effects. Also, release of calcium ions can induce hard tissue formation and prevent resorption. The released calcium ions react with phosphorous in tissue fluids and form hydroxyapatite [ 24 ]. Considering the effective role of pH rise and presence of calcium ions in prevention or management of external root resorption, this study sought to assess the pH alterations of the periradicular area within 3 months following the application of CH and MTA as intracanal medicaments. The null hypothesis was that no significant difference would be found between the CH and MTA groups regarding the pH alterations at different time points. 

## Materials and Method

This *in vitro*, experimental study evaluated 45 sound human maxillary central incisors extracted for purposes not related to this study. The study was approved by the Ethics Committee of School of Dentistry, Islamic Azad University, Khorasgan Branch (IR.IAU.KHUISF.REC. 1397.245). The sample size was calculated to be 45 teeth using the Cochran’s formula assuming the maximum error of 0.34, alpha=0.05, beta=0.2, d=0.34, 95% confidence interval, and study power of 80%. The inclusion criteria were mature human maxillary central incisors with complete apices, no root curvature, one single canal, mean root length of 16 mm, absence of root cracks, root fracture or root caries, no cervical wear, and no previous restoration.

The selected teeth were cleaned with a soft prophy brush and immersed in 2% sodium hypochlorite solution for 30 min. After taking a periapical radiograph, the crowns were cut at the cementoenamel junction using a diamond disc under water spray such that 16 mm of root length remained. The pulp tissue was removed using a barbed broach. A #15 K-file was used to determine the working length. The file was introduced into the canal until its tip was visible at the apex; 1mm of this length was subtracted to determine the working length. The canals were filed to #60 using the step-back technique. The coronal part of the canal was shaped using #1 to #3 Peeso reamers (Mani, Japan). The canals were passively irrigated with 2 mL of 5.25% sodium hypochlorite between filings using a 27-gauge needle. Next, defects measuring 3×3mm in diameter and 1mm in depth were created in the middle third of the roots. For smear layer removal, the canals and the defect sites were rinsed with 5.25% sodium hypochlorite (Cerkamed, Poland) for 1 min and 17% EDTA (Ariadent, Iran) for 1 min followed by a final rinse with 5.25% sodium hypochlorite for 1 min. The entire root surface, except for the defects, was sealed with two layers of nail varnish. Each root was stored in 10 mL of saline for 24h. Next, the teeth were randomly divided into two experimental groups (n=20) and one control group (n=5). 

In group 1, MTA (Angelus, Brazil) was mixed with distilled water in 3:1 ratio according to the manufacturer’s instructions and delivered into the canal using a MTA carrier. It was then condensed using a moist cotton pellet and a hand plugger (Mani, Japan). 

In group 2, CH powder (Merck, Germany) was mixed with distilled water according to the manufacturer’s instructions to obtain CH paste with a powdery consistency. It was then delivered into the canal using an amalgam carrier and condensed with a paper point. A periapical radiograph was obtained to ensure optimal packing and filling of canals. The negative control root canals were filled with distilled water. The coronal region was sealed with self-cure glass ionomer (GC, Japan), one layer of sticky wax and two layers of nail varnish (Flomar, Italy). Next, the teeth were immersed in 8 mL of saline in a glass container and the container was incubated at 37°C. All teeth were stored in saline and the pH was measured at 1 and 2 weeks, and 1 and 3 months, using a pH meter (Oakton, Malaysia). To simulate the *in vivo* dynamic state, the solutions were refreshed after each time of measurement to prevent the accumulation of ions. The device was first calibrated prior to measurements. Next, the sensor was cleaned with distilled water and placed in the solution to measure the pH. The sensor remained in the solution until the displayed value was stabilized. The value was recorded. Data were analyzed using SPSS version 22 (SPSS Inc., IL, USA) via one-way ANOVA, repeated measures ANOVA, Tukey’s post-hoc test and Bonferroni test. 

## Results

Table 1 shows the mean pH in the three groups of distilled water, MTA and CH at 1 and 2 weeks, and 1 and 2 months. One-way ANOVA revealed that the mean pH was significantly different among the three groups at 1 and 2 weeks (*p*< 0.001) but this difference was not significant at 1 (*p*= 0.20) or 3 (*p*= 0.52) months. The Tukey’s post-hoc test revealed that the mean pH in the CH group was significantly higher than that in the MTA group and the mean pH in the MTA group was significantly higher than that in the control group at 1 and 2 weeks (*p*< 0.05).

**Table1:** Mean pH in the three groups of distilled water, MTA (Mineral Trioxide Aggregate) and CH at 1 and 2 weeks and 1 and 2 months

Time	Group	Mean	Std. deviation	Minimum	Maximum	*p* Value
1 week	Distilled water	7.20	0.08	7.12	7.28	<0.001
	MTA	7.84	0.20	7.63	8.09	
	CH	8.30	0.28	8.03	8.75	
2 weeks	Distilled water	7.20	0.05	7.15	7.25	<0.001
	MTA	8.86	0.29	8.58	9.22	
	CH	9.34	0.59	8.61	10.13	
1 month	Distilled water	7.20	0.06	7.14	7.26	0.20
	MTA	7.70	0.51	6.96	8.30	
	CH	7.54	0.23	7.24	7.86	
3 months	Distilled water	7.20	0.04	7.18	7.23	0.52
	MTA	7.41	0.31	6.96	7.71	
	CH	7.32	0.11	7.19	7.48	

Repeated measures ANOVA showed a significant difference in the mean pH in the MTA and CH groups at different time points (*p*< 0.001). The Bonferroni post-hoc test revealed that in the MTA group, the mean pH at 2 weeks was significantly higher than that at other time points (*p*< 0.05). However, the difference in this respect was not significant between other time points (*p*> 0.05). In the CH group, the mean pH at 2 weeks was significantly higher than that at 1 week, and 1 and 3 months (*p*< 0.05). But, the difference in the mean pH was not significant between 1 and 3 months (*p*> 0.05). 

Maximum pH was noted in the CH group at 2 weeks while minimum pH was noted in the CH group at 3 months. The mean pH was maximum at 2 weeks in both CH and MTA groups. At 1 and 3 months, the mean pH decreased in both CH and MTA groups compared with the values at 2 weeks; however, this reduction was greater in the CH group than the MTA group but not significantly (*p*= 0.52).

## Discussion 

This study assessed the pH alterations of the periradicular area within 3 months following the application of CH and MTA as intracanal medicaments. Our methodology in this study was adopted from similar previous studies [ 25- 26]. The results showed that the mean pH was in its maximum value at 2 weeks in both the MTA and CH groups. At 1 and 3 months, the mean pH decreased in both CH and MTA groups compared with the values at 2 weeks. Considering the refreshment of the solution after each measurement, the magnitude of pH reduction (difference from the neutral pH) gradually decreased over time. Since the washout rate of CH is considered constant, by refreshing the solution, accumulation of ions is prevented. Thus, it is logical that the pH reduction at 2weeks (compared with the neutral state) is smaller than that at 1 week. Also, in longer periods of time, the chances of washout of CH are higher (due to prolonged time period). Thus, pH measurement at 1 and 3 months revealed greater magnitude of pH reduction compared with 2 weeks. Over time, the mean pH in the MTA group remained higher than that in the CH group, but not significantly. Reduction in pH in the CH group, compared with the MTA group, can be attributed to CH washout (although in small amounts) [ 14]. The pH rise and its stability in the MTA group at 1 and 3 months can be attributed to the long setting time and optimal dimensional stability of MTA. It should be noted that we measured the pH of the solution in which, the roots had been immersed, and could not measure the pH of root surface due to the unavailability of micro pH meter. Considering the significantly higher pH value of the CH group at 1 and 2 weeks compared with the MTA group, the null hypothesis regarding absence of a statistically significant difference between the CH and MTA groups was rejected in the first 2 weeks. However, the null hypothesis was accepted at 1 and 3 months due to the absence of a significant difference in pH between the two experimental groups at these time points. It should be noted that the pH values measured in our study were significantly higher than the values reported in other studies. Farhad et al. [ 27] evaluated the pH alterations and release of calcium ions in periradicular area at 24, 48 and 168 h following the use of CH, ProRoot MTA and MTA Angelus. They concluded that the concentration of calcium ions and the mean pH in the periradicular area increased in all three groups at 1 week. On the other hand, the mean pH in the CH group was significantly higher than the values in the two MTA groups at all time points but MTA Angelus and ProRoot MTA were not significantly different in this respect [ 27]. Their results were in line with our findings. However, we evaluated the pH over a longer period of time and showed that MTA could maintain the pH high for a longer period of time. Duarte et al. [ 24] evaluated the pH and calcium ion release from MTA Angelus and ProRoot MTA for root filling and perforation repair at different time points. They found that the calcium ion release and pH increased in both groups early after the study onset. However, they noticed a descending trend over time and the pH and calcium ion release in the MTA Angelus group were greater than the corresponding values in the ProRoot MTA group. Their results regarding pH rise were in agreement with our findings at 1 and 2 weeks. Sáez et al. [ 28] measured the mean pH and calcium ion release around the roots at 7, 30 and 60 days following the application of CH and MTA. They concluded that the pH increased in CH group at all time points. They found no significant difference between the experimental and control groups at 30 days, and maximum pH was noted in CH group at 60 days [ 28]. Their results regarding no significant difference in the mean pH at 30 days were in line with our findings. However, their results regarding maximum pH of CH group at 60 days were different from our findings. They did not measure the pH at 2 weeks, which may explain the difference in the results. Misra et al. [ 29] measured the pH and calcium ion release from CH at different time points by spectrometry. They found maximum pH in the CH group at 30 days. Their results regarding the mean pH were different from our findings. This controversy in the results can be attributed to the different CH solvents used in their study, method of measurement, experimental setting, and duration of study. Fuss et al. [ 30] evaluated the pH alterations of distilled water in which, roots filled with CH had been immersed. They concluded that the pH alterations were minimal within the first 10 days, which was different from our findings. In general, different types of materials used, method and time of measurement, testing environment, and duration of study can explain difference in the results. Future studies over longer periods of time are required to assess calcium ion release from intracanal medicaments. Calcium ion release and the mean pH of different types of MTA should also be compared over longer periods of time. 

## Conclusion

Within the limitations of this in vitro study, it may be concluded that CH may be preferably used as intracanal medicament in the first weeks following the initiation of root resorption to provide a high pH at the site. Afterwards, MTA can be applied to maintain the pH high for a longer period of time without the need for medicament exchange. CH and MTA can both effectively increase the pH of the periradicular area. 
